# Physical environmental conditions determine ubiquitous spatial differentiation of standing plants and seedbanks in Neotropical riparian dry forests

**DOI:** 10.1371/journal.pone.0212185

**Published:** 2019-03-13

**Authors:** Alejandra De León Ibarra, Néstor A. Mariano, Valentino Sorani, Gabriel Flores-Franco, Evodio Rendón Alquicira, Elisabet V. Wehncke

**Affiliations:** 1 Centro de Investigación en Biodiversidad y Conservación, Universidad Autónoma del Estado de Morelos, Cuernavaca, Morelos, México; 2 Instituto de Ambiente de Montaña y Regiones Áridas, Universidad Nacional de Chilecito, Chilecito, La Rioja, Argentina; Fred Hutchinson Cancer Research Center, UNITED STATES

## Abstract

Mexican tropical dry forests are remarkably extensive and floristically diverse despite manifesting alarming rates of deforestation. Riparian habitats within dry forests provide critical ecological benefits that may mitigate negative impacts, but processes underlying riparian functions are still not well understood. We identified physical environmental conditions affecting the composition and abundance of standing vegetation assemblages and woody and herbaceous components in soil seedbank assemblages of riparian corridors in central Mexico using mainly NMDS ordination techniques, permuted analysis of variance (PERMANOVA), permuted analysis of multivariate dispersions and constrained ordination (CAP). We then determined representative species associated with particular environmental conditions using an indicator species analysis and assessed the effects of physical environmental variables/factors on total seed abundances by fitting a mixed-effect model. For the standing vegetation study, we assessed the effects of the type of the river condition (differing in surface flow permanence), location, and height above river level on the community composition based on three species importance criteria (abundance, coverage and DBH). For the soil seedbank study, we assessed the effects of these variables/factors plus season and land use. Spatial heterogeneity was a prevailing feature in riparian vegetation, in the standing vegetation and soil seedbank of both woody and herbaceous components. Height above river level had a significant effect on the three species importance criteria of standing vegetation and so did the interaction between surface flow permanence and height on coverage. The soil seedbank of woody and herbaceous plants showed significant differences between seasons; *Taxodium mucronatum* was an indicator tree species in dry seasons. Land use, height, surface flow permanence and the interaction between land use and surface flow permanence had significant effects on the soil seedbank of herbaceous plants. Total seed abundances in the soil varied between years and were higher at lower height values, during the dry seasons, and when rivers were permanent. Tree communities, commonly the most important elements in riparian ecosystems, were preserved in the soil seedbank of cultivated areas for >30 years. Seeds of herbaceous communities were predominant and ecologically relevant as indicator species because of their high sensitivity to several key environmental factors, constituting a critical component of Mexican tropical dry forests riparian corridors.

## Introduction

Riparian ecosystems are very striking from an ecological point of view because they constitute ecotones between terrestrial and aquatic systems and therefore represent areas where many physical, abiotic and biotic processes are continuously changing and transforming [1]. These corridors are subject to important spatio-temporal dynamics mainly directed by variable flow regimes [2,3] and the arrival of propagules through hydrochory providing high resilience for riparian plant communities and a regular turnover of seeds [4]; and it appears to be poorly related to the dynamics of the soil seed bank, the temporal storage of propagules in riparian soils [5]. Several studies have shown that vegetation along rivers is ecologically dynamic in space and time and produces critical ecological benefits that mitigate the impacts of anthropogenic activities [6,7]. Riparian vegetation limits erosion by regulating flow velocity and magnitude, surface runoff, and sediment loading, while increasing aquifer recharge and infiltration [8,9,10,11]. It also modifies evapotranspiration, precipitation uptake, soil moisture retention [8,11,12,13] and freshwater biological activity [e.g., 14,15]. In the context of landscape, it also has an indirect impact on the stability and quality of surrounding land [16]. Throughout history, humans have settled at these locations because of their proximity to essential resources such as water and fertile land, but also for recreation and spirituality [17,18]. Over time, human activity and land use conversion to agriculture and grazing have led to loss of natural vegetation, seedbanks and biodiversity [19,20,21]. Stream alteration, agriculture and logging in and around riparian zones severely threaten their integrity, functionality and sustainability [17,22,23,24], and it has been estimated that 65% of river habitats in the world are threatened by land use change [25].

Tropical dry forests are among the most extensive and floristically rich ecosystems in tropical habitats [26,27,28,29]. They also constitute the most endangered tropical ecosystem globally [29,30]. While Mexican tropical dry forests have remarkably high floristic diversity and levels of endemism, they suffer from alarmingly high rates of deforestation and lack of effective long-term plans for their management and conservation [30]. Recently, it has been pointed out that seed dispersal patterns generated by rivers are significant mechanisms for structuring the composition and distribution of the riparian plant community in Mexican tropical dry forests [31], suggesting that their conservation potential for restoration needs to be considered. This is even more critical given that tropical dry forests is the most extensive and best-represented tropical ecosystem in Mexico, occupying ca. 14% of the country, and 266,000 km^2^ of the Pacific slope [32,33].

Despite the key role played by tropical dry forests riparian areas, the processes, drivers, and dynamics of soil seedbanks and standing vegetation are not well understood, much less their relationships with the abiotic conditions of these ever-changing and vulnerable corridors. One of the main abiotic conditions that could affect processes that maintain riparian vegetation functions is river regime [1,34], which describes the river’s small and large flow fluctuations, as well as its surface flow permanence (temporality or permanence of running water along a river throughout the year). Temporary rivers do not maintain a continuous flow of water year-round; even so, isolated pools of water may remain throughout the dry season, offering storage for a large quantity of nutrients during dry periods [35] and generating different patterns of vegetation distribution [36]. These pools are often the only places in the watershed with sufficient soil moisture to maintain a sizeable plant community [37] and have thus been suggested as hotspots of plant diversity and abundance compared to their watersheds [38,39]. They may also function as oases [40], as they attract small and medium vertebrates [41] which may in turn provide food for larger predators that enter these areas [42]. In contrast, permanent rivers maintain water all year round, fed by surface and/or groundwater [43], and some studies have shown that sites with permanent flows during years with less frequent rain are richer and more diverse than sites with seasonal flow [36]. Thus, the effect of river seasonality on plant richness and diversity is not easily predicted.

The effects of the physical environment on the composition of the riparian soil seedbank is a subject that deserves attention because it constitutes a very important way in which freshwater ecosystems contribute to the diversity of riparian plant communities [16,44]. Riparian soil seedbank composition is influenced by the natural dispersion of seeds [44,45], and is mainly affected by precipitation [36], river flows [46,47,48,49], and disturbance [50,51,52]. Removal of seeds due to torrential rains or to flood events along corridors is common, particularly in dry environments [53]. Even if seeds can be dispersed over great distances, their accumulation in the SSB and subsequent emergence, establishment and growth may depend on diverse environmental factors such as soil type, light, degree of site alteration, slope and distance to the river [54,55,56], as well as the ability of the riverbank to trap waterborne diaspores [57]. Natural variability of river flows (floodings and small fluctuations) is of utmost importance for sustaining the ecological integrity of riverine ecosystems [12,48], and it has been shown to be determinant in the kind of seed assemblage and numbers transported by the river in tropical dry forests of central Mexico [58]. River overflows may eventually affect sites of silt accumulation and deposition, or on the contrary, may produce erosion and physical damage to new seedlings, affecting standing vegetation [59]. Additionally, anthropogenic disturbance of riparian corridors or adjacent lands could promote the establishment of invasive species, which in turn may cause changes to the structure and function of riparian systems [50]. It has been shown that the effects of intense agricultural land use on riparian habitats are particularly important [16] and may negatively affect the riparian soil seedbank. For example, Dalton et al. [60] compared the riparian soil seedbank across a large temperate watershed and along a gradient of agricultural land use in Ontario, Canada, and found that loss of natural habitat and nutrient enrichment had negative effects on the riparian soil seedbank community composition. Thus, in boreal/temperate ecosystems it has been shown that landscape scale processes (water flow and hydrological connectivity as key drivers of ecological integrity of rivers) largely influence the structure of spatially riparian plant communities [3], however local processes can also control patterns of riparian species richness [61].

The main aims of this study were threefold; 1) to investigate which physical environmental conditions (height above river level, season, river condition related to surface flow permanence, site, and land use) affect the composition of the standing vegetation assemblage and the composition and abundance of the soil seedbank of both woody and herbaceous plant assemblages, 2) to determine which species, if any, are representative of a particular environmental condition, and 3) to assess the effect of physical environmental variables on total seed abundances. We expected to find a significant effect of the physical environment, in particular the location, height above river level, and surface flow permanence (temporary/permanent), on the abundance, diameter at breast height (DBH) and coverage of the standing vegetation and the soil seedbank community. We also expected to find significant effects of season (dry/rainy) and land use (cultivated/natural vegetation areas) on the diversity and abundance of the soil seedbank community. Because species have developed unique characteristics related to their life histories and to their relationships with the environment in order to maximize their reproductive success [62,63], we expected the effects of environmental variables on species distribution patterns to differ between standing vegetation and soil seedbank assemblages of woody and herbaceous vegetation. The study was performed on the riparian vegetation along six tributaries to the Amacuzac River in central Mexico, running through a mountainous landscape within a mixture of agricultural and natural tropical dry forests areas. In identifying the effects of key abiotic conditions and anthropic perturbation on Mexican riparian tropical dry forests vegetation, this study aims to contribute to explaining patterns of diversity and dynamics of riparian corridors in tropical dry forests habitats, which in turn, will contribute to elucidate the potential role of tributaries for management and conservation action at local and at the basin scale.

## Methods

### Study area

The Amacuzac is one of the most important rivers of the Balsas drainage basin and one of the largest rivers in southern Mexico. It originates in the foothills of the volcano Nevado de Toluca, at an altitude of 2600 masl and flows into the Pacific Ocean (SPP 1988; CNA 1998). The Amacuzac is the largest river basin in southern Morelos, Mexico, running through 80 km of the state (coordinates between 18°39’ and 18°19’ N, and 99°28’ and 99°03’W) and traversing elevations ranging from 1142 to 712 masl. It crosses a mixture of agricultural and tropical dry forests areas characterized by a landscape of deciduous vegetation [64] and is composed of both temporary and permanent streams (IMTA 2016, accessed: January 2016, https://www.gob.mx/imta).

**Fig 1:**
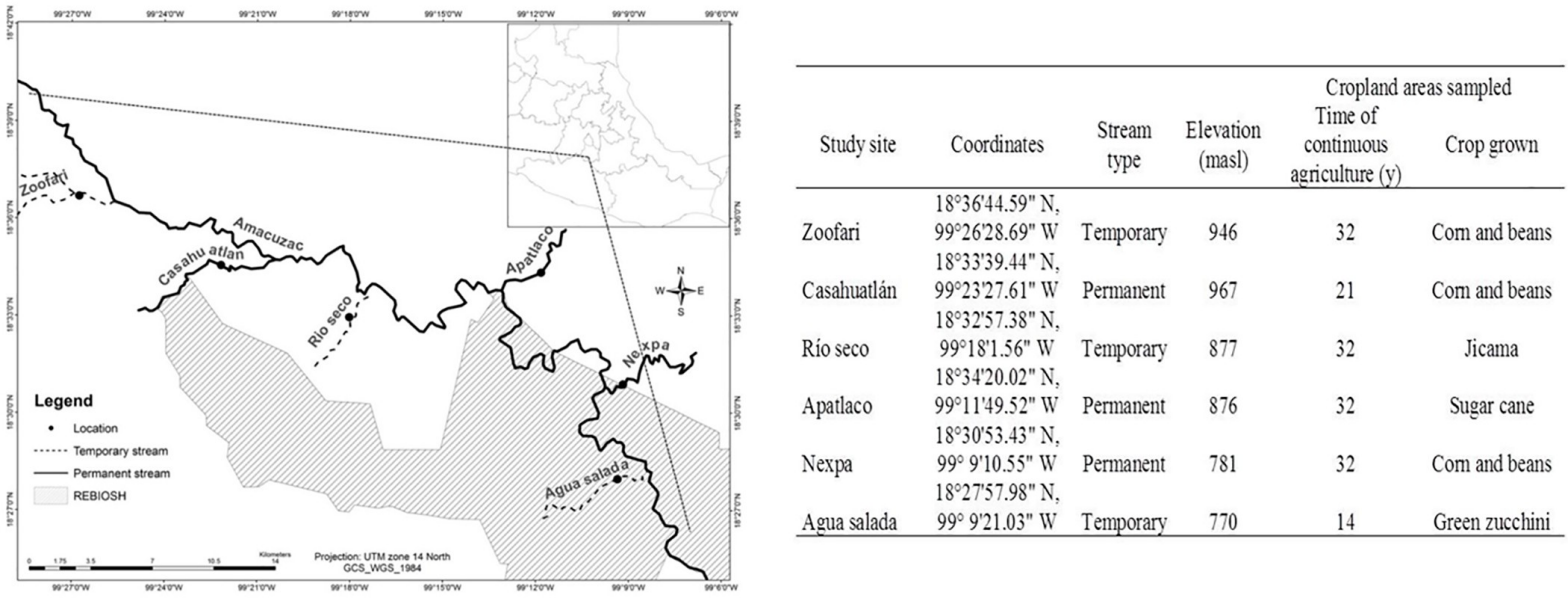
Map and general information of study areas. Location and general characteristics of study sites: Apatlaco, Casahuatlán and Nexpa are permanent streams; Zoofari, Agua Salada and Rio Seco are intermittent streams. Modified and republished with permission from INEGI (Mexico) (Terms of Free Use of INEGI Information: http://www.beta.inegi.org.mx/inegi/terminos.html).

The study was carried out over a period of two years (2015–2016) along six tributary streams that differed in surface flow permanence (three temporary and three permanent) to the Amacuzac River (Fig 1). We defined permanent and intermittent streams respectively, as those in which surface flow was present 100% of the time and 26–95% of the time [65]. The climate is seasonal, hot subhumid with summer rains from June to October and a dry season from November to May. Mean annual temperature is 21.5°C [66] and annual precipitation ranges between 900 and 1500 mm [67,68]. The locations with permanent streams were: ‘Nexpa’, ‘Apatlaco’ (one of the most contaminated streams in the State), and ‘Casahuatlán’. Locations with temporary streams selected were: ‘Zoofari’, ‘Río Seco’, and ‘Agua Salada’ (a groundwater-dependent ecosystem). A map of the study areas is shown in Fig 1. The mean (± 1 sd) linear distance between locations was 8.3 ± 2.1 km (range: 5.37 to 11 km).

### Experimental design

#### Vegetation and soil sampling

Along each of the six tributaries 3 km upstream of where the tributary met the Amacuzac River, we established six transects 50 x 2 m, perpendicular to the river; (three in cropland and three in natural vegetation areas), separated from each other by 50 m. In all cropland sites sampled the practice is to fallow (to till the land without sowing it later, so that it rests, is aerated and enriched), only one of them, Apatlaco, burns the cultivation area. Crop type grown differed between sites (Fig 1). We characterize standing vegetation in each transect in natural vegetation areas, counting and identifying all trees and shrubs exceeding 1 meter in height and with a diameter at breast height (DBH) greater than 1cm and measured their DBH and crown coverage. All measurements were carried out by one observer and this was the same person who took the measurements for all trees along transects (strips of 50 x 2 m) by defining the true vertical projection of the canopy. Thus, for every tree species within a canopy layer, crown boundaries were vertically projected onto transects. The distance along a transect line that the crown intercepted was recorded. For each tree the average diameter was calculated and this measure was incorporated into the formula of canopy area (A) using the formula of an ellipse.

*A = π ÷ 4 × D*^2^, where D is the average canopy diameter.

This study was carried out on private land, so owners of the land gave permission to conduct the study on these sites. We also confirm that the field studies did not involve endangered or protected species. To characterize the soil seedbank composition, we collected five soil samples (10 cm wide x 10 cm long x 10 cm deep) from each of the aforementioned transects at 10, 20, 30, 40 and 50 m from the river. We sampled in this way during the rainy and dry seasons of two consecutive years, totaling 720 soil samples. The soil seedbank composition was thus determined using the seedling emergence method over a 3-mo period [69,70], under greenhouse conditions immediately after each sampling period (May and November of 2015–2016, corresponding to rainy and dry season). After removing larger branches, leaf litter, and rocks, soil samples were deposited in a greenhouse and germinated on a mixture of substrates (Agrolite:Peetmoss 20:30) in labeled perforated trays. Trays were watered for five minutes, twice a day and were checked weekly for seedlings in order to identify species.

#### Characterization of environmental variables and factors

We considered the ‘distance to the river’ as the distance from the river to the closest point on the transect; ‘slope’ was measured every 10 m along each transect. With these measurements we calculated ‘height’ as h = sin of the slope (degree) x distance (m). Height was thus defined as the topographic level relative to the river level. The ‘season’ (Se) was either dry or rainy, ‘surface flow permanence’ (SFP) refers to the river condition, in other words, whether the presence of running water in the stream is present only seasonally (temporary stream) or year-round (permanent stream) and ‘site’ refers to each particular study location. Finally, ‘land use’ (LU) was a two-level categorical variable referring to whether each site was cultivated or natural vegetation.

### Data analysis

Richness and abundance were calculated for standing vegetation and soil seedbank considering surface flow permanence (permanent/temporary), seasons (dry/rainy), and land use (natural vegetation/crop areas). We calculated the species importance value index (IVI; developed by Curtis & McIntosh [71], and applied by Cintrón & Schaeffer-Novelli [72], Corella et al. [73] and Zarco-Espinosa et al. [74], among others) for standing vegetation and for the soil seedbank. IVI = RF + RDe + RD, where RF is the relative frequency, RDe is the relative density and RD corresponds to the relative dominance. Relative frequency per site refers to the proportion of individuals for each physical environmental condition considered (distance to the river, slope, season, surface flow permanence, site, and land use) in the forest containing the given species x 100%; relative density was calculated as the number of individuals of a species/sampled area/total density x 100%; relative dominance was the basal area of a species/sampled area/total dominance x 100%.

#### Statistical analyses

Non-metric multidimensional scaling (NMDS) ordinations on Bray-Curtis dissimilarities, permuted analysis of variance (PERMANOVA) and permuted analysis of multivariate dispersions were performed using the functions metaMDS, adonis and betadisper, respectively from the vegan package in R [75,76]. Canonical analysis of principal coordinates (CAP) implemented through the capscale function in vegan was used to perform partial constrained ordinations in order to test for linear relationships between height and both, standing vegetation and seed bank community composition (Bray-Curtis dissimilarities). The statistical significance of height above river level was assessed by restricting permutations within each transect considering spatial positions on it and at the same time, conditioned by transect id, nested within site. All permutation tests were performed with 999 permutations. To test for unequal dispersion of variability among groups, permutational multivariate analyses of dispersion were conducted for all significant PERMANOVA outcomes.

#### Effects of physical environmental variables on standing vegetation

For the standing vegetation we evaluated the effect of height above river level, surface flow permanence and the interaction surface flow permanence x height by using three quantitative matrices of species attributes containing information on abundance, DBH and coverage. These measurements were selected because they provide complementary information on community attributes. Although all are related to the use of habitat resources, abundance may correlate poorly with biomass (indirectly expressed by DBH and coverage). In turn, DBH and coverage are complementary measurements, whose correlation varies depending on tree architecture as a function of the crown diameter/height ratio (i.e., tree crowns with high diameter/height ratios may have the same DBH as trees with low diameter/height ratios). Values of abundance and coverage (expressed as m^2^/transect surface) were square-root transformed and DBH (expressed as cm^2^/transect surface) was transformed using natural logrithm (1 was added to all values in order to yield only positive transformed values).

#### Effects of physical environmental variables on soil seed banks

A permutational multivariate analysis of variance (PERMANOVA) hierarchical design was used to test for significant differences in seedbank community affected by surface flow permanence and season by including these factors (surface flow permanence and season) and their interaction term (season x surface flow permanence). Another hierarchical design was used to test for significant differences between cultivated and natural vegetation areas and their interaction with surface flow permanence (LU x SFP). In both analyses, surface flow permanence was considered as the upper factor of the hierarchy design. A restriction of our analyses was that, when analyzing seedbank compositions the occurrence of “empty” cases with no data on some rows of the matrix (i.e., “empty” position/samples) precluded performing hierarchical analyses with the original data matrix. In order to fix this problem and to evaluate the effect of land use, season and their interaction with surface flow permanence, abundances were combined within transects. We then evaluated the effect of surface flow permanence (temporary/permanent) by permuting sites whilst the order of transects were retained within sites. After fitting the above general models we extended our analysis by performing separated analyses on natural and crop areas. By this procedure our intention was to provide a deeper and more detailed comprehension of the impacts of environmental factors and variables on riparian seedbank communities. Abundances were square-root transformed. Herbaceous species containing < 3 individuals were not included in the analysis.

The matrix used in all analyses was the result of adding the number of seeds recorded from two years. Bray-Curtis similarity matrices were visualized using non-metric multidimensional scaling (NMDS) plots.

We performed the Indicator Species analysis [77] to determine which species are significantly representative of a particular environmental condition [78]. We used the function ‘multipatt’ in the package ‘indicspecies’ [79], of the R statistical computing program [80]. This procedure is an extension of the original Indicator Value Index (IndVal) estimation and measures the association between species and particular environmental/site groups [78,79]. We tested the statistical significance of this relationship using a permutation test.

Finally, to assess the effect of the particular environmental condition on total soil seed abundances, we fitted a mixed-effect model. The fixed-effects included in the model were ‘year’ (Yr), ‘season’ (Se), ‘surface flow permanence’ (SFP), ‘land use’ (LU) and the interaction between Se, SFP and LU (all factors), and ‘height above river level’ (h) was included as covariate, as well as the h x Se and h x SFP interaction terms. The random component of the model had a hierarchical nesting structure, with ‘transect identity’ (T) nested within LU, nested within ‘site’ (SL); and Yr was used as the intercept. As we did with the multivariate analysis, after adjusting the general model, we performed separated analyses on natural and crop areas.

Seed abundance values + 1 were log-transformed to meet model assumption of homocedasticity and normality of residuals. We report mean and standard error values for untransformed data. We fitted linear mixed-effects models using the ‘lmer’ function of the ‘lme4’ package [81], of the R statistical computing program. To find the best (simplest) model we used the ‘step’ function of the ‘lmerTest’ package [82] which selects the final model by using a backwards comparison starting with the most complex model (including all terms and specified interactions) and arrives at the final (simplest) model by retaining only those terms that are statistically significant (P < 0.05).

## Results

### Standing vegetation composition: Trees and shrubs

We found a total of 52 woody species along the tributaries of the Amacuzac River (S1 Table), and a total abundance of 270 individuals (Mean ± 1 s.e. per transect = 15 ± 1.2, n = 18), distributed over 1800 m^2^. This corresponds to approximately 1500 individuals/ha. Of the total species, 77% were trees and 23% shrubs. The sum of DBH measures of woody species represented 13.4% of the sampled area (13.4 ± 5.1; ~ 1340 m^2^ /ha), while the sum of tree coverages represented 4.7 times the sampled area (4.74 ± 0.8). Of the total woody species, 36 were found exclusively at temporary streams, 32 at permanent streams, and 15 at both (S2 Table); 49 (94%) were native and 3 (6%) were exotic species (S1 Table). The tree species with the highest importance values (IVI) in the standing vegetation were *Ficus cotinifolia*, *Acacia cochliacantha* and *Tabernaemontana litoralis* (S1 Table).

### Effects of physical environmental variables on standing vegetation

**Fig 2:**
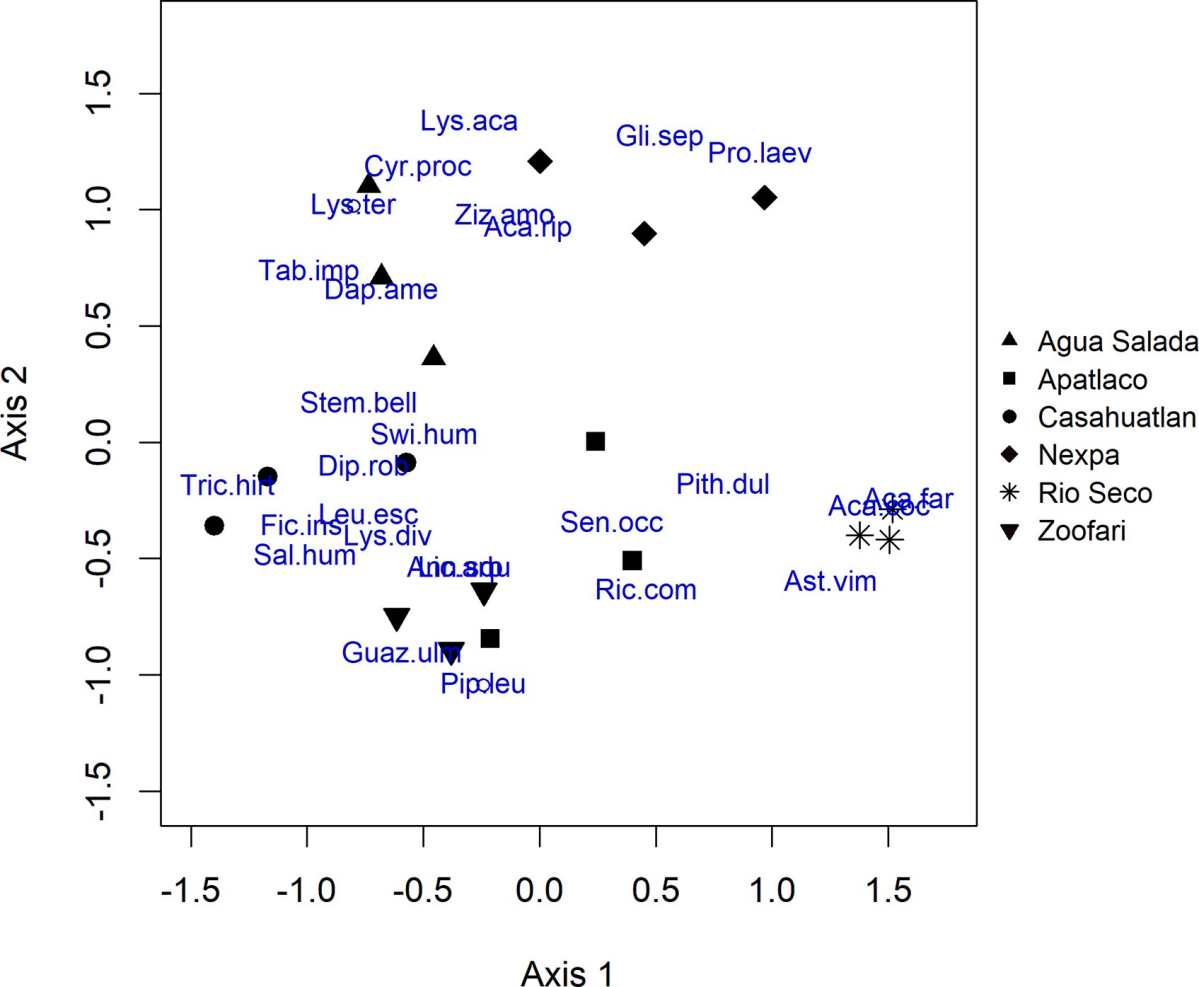
Spatial heterogeneity of the standing vegetation community along six tributaries/sites to the Amacuzac River in Morelos. Figure showing results of the NMDS ordination of the standing vegetation community. Species are included in the ordination. References for species: Aca.coc: *Acacia cochliacantha*, Aca.far: *Acacia farneciana*, Aca.rip: *Acacia riparia*, Ann.squ: *Annona squamosa*, Ast.vim: *Astianthus viminalis*, Bur.gran: *Bumelia optusifolia*, Cyr.proc: *Cyrtocarpa procera*, Dap.ame: *Daphnopsis americana*, Dip.rob: *Diphysa robinoides*, Fic.ins: *Ficus insipida*, Gli.sep: *Gliricidia sepium*, Guaz.ulm: *Guazuma ulmifolia*, Inga.spu: *Inga spuria*, Leu.esc: *Leucaena esculenta*, Lic.arb: *Licanea arborea*, Lys.aca: *Lysiloma acapulcensis*, Lys.div: *Lysiloma divaricata*, Lys.ter: *Lysiloma tergemina*, Pip.leu: *Piper leucophyllum*, Pith.dul: *Pithecellobium dulce*, Pro.laev: *Prosopis laevigata*, Ric.com: *Ricinus communis*, Sal.hum: *Salix humboldtiana*, Sen.occ: *Senna occidentalis*, Stem.bell: *Tabernaemontana litoralis*, Swi.hum: *Swietenia humilis*, Tab.imp: *Tabebuia impetiginosa*, Tric.hirt: *Trichilia hirta*, Ziz.amo: *Ziziphus amole*.

NMDS ordination showed clustering of woody species assemblages by site and PERMANOVA analyses revealed that woody communities were significantly different between sites (pseudo-F = 5.84, p < 0.001); the same consistent pattern emerged for the three matrices using abundance, DBH and coverage for vegetation measures (Table 1, Fig 2). Among environmental variables, CAP analysis showed that height above river level had a significant effect on the standing tree community composition (Table 1). Surface flow permanence had no effect on the riparian standing tree community (Table 1). The interaction Surface flow permanence x height was not significant either for abundance and DBH, except for coverage (Table 1).

**Table 1:** Effects of height above river level, surface flow permanence (temporary/permanent), and the interaction between surface flow permanence and height, on the standing vegetation community. Effects on three measurements of species importance (Abundance, DBH and Coverage) were evaluated. Significant effects are highlighted in bold.

Species importance parameters	Abundance ^(1)^		DBH		Coverage	
Variables	F^(1)^	P	F	P	F	P
Height	2.23	**0.036**	2.76	**0.012**	1.95	**0.034**
**Factors**						
Surface flow permanence (temporary/permanent)	4.61	0.896	5.98	0.730	9.17	0.112
SFP x Height	2.12	0.550	2.60	0.314	3.11	**0.032**

(1) Pseudo F

### Woody and herbaceous soil seed bank composition

We were able to identify 10033 individuals, corresponding to 142 species from the soil seedbank at all study sites, of which 8% were trees, 3% shrubs and 89% herbs (S3 Table). Of the total species, 87 were native (61%), 30 exotic (21%), and 25 uncertain (18%) (S3 Table). We found 131 species (3109 individuals) at temporary streams and 161 species (6924 individuals) at permanent streams, a difference which may be due to the herbaceous component of the soil seedbank. A total of 135 species were found at both temporary and permanent streams. We found 161 species in natural vegetation areas, 136 in crop areas, and 115 in both, independent of stream seasonality or season. Soil seedbank samples corresponding to the two dry seasons contained 158 species (6254 individuals), those corresponding to the rainy seasons contained 120 species (3779 individuals), and 103 species were found during both seasons, independent of the type of stream or land use.

### Effects of physical environmental variables on soil seed bank

#### Effects on the woody soil seed bank assemblage

**Fig 3:**
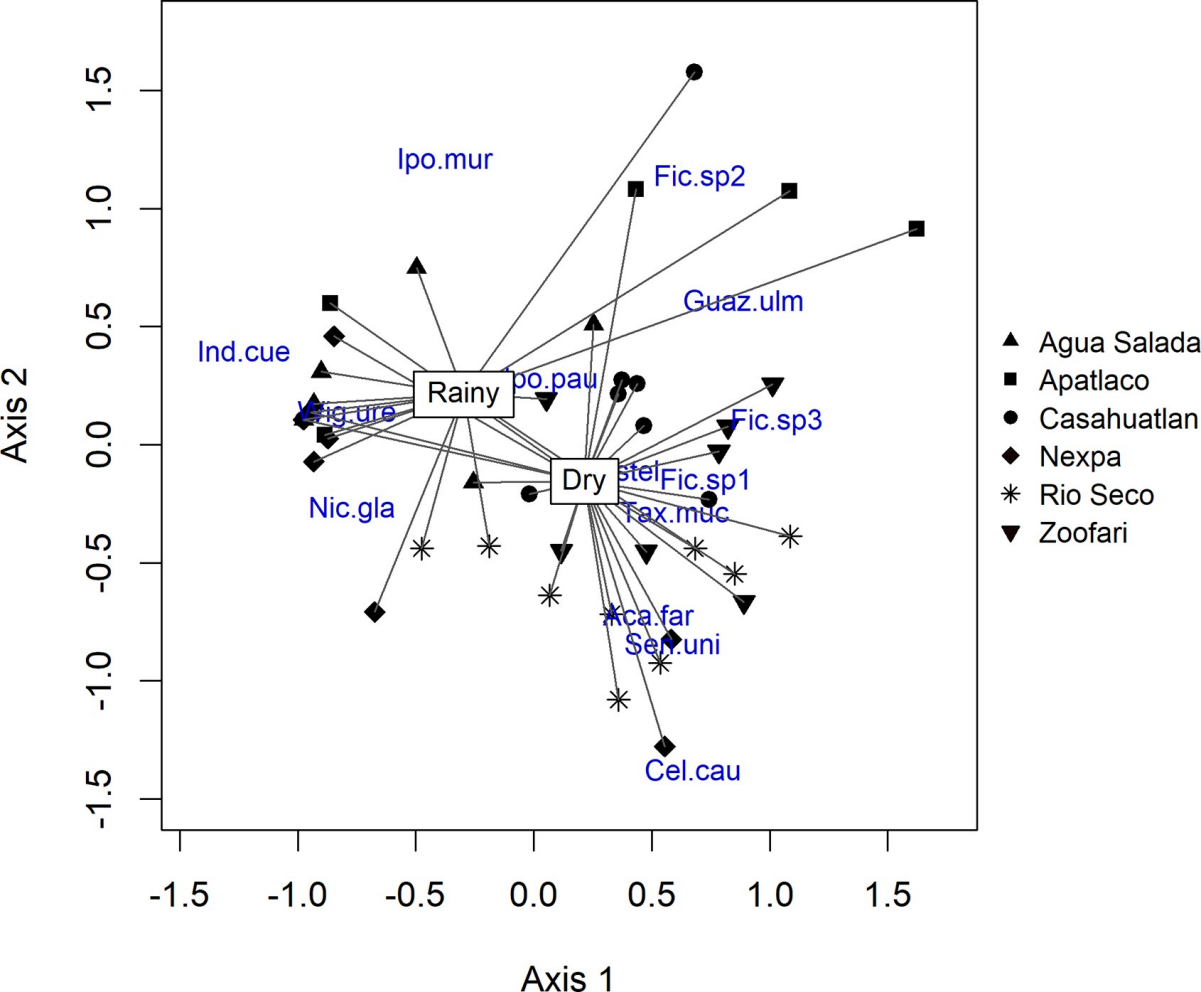
Spatial and temporal heterogeneity of woody assemblages in the soil seedbank in six tributaries/sites to the Amacuzac River in Morelos. Figure showing spatial variation, represented by differences between sites and temporal variation, represented by differences between dry and rainy seasons of woody species assemblages in soil seedbanks, using NMDS ordination. References for species: Aca.far: *Acacia farneciana*, Cel. cau: *Celtis caudata*, Fic.sp1: *Ficus* sp1, Fic.sp2: *Ficus* sp2, Fic.sp3: *Ficus* sp3, Guaz.ulm: *Guazuma ulmifolia*, Ind. cue: *Indigofera cuernavacana*, Ipo. mur: *Ipomoea murucoides*, Nic. gla: *Nicotiana glauca*, Sen. uni: *Senna uniflora*, Tax muc: *Taxodium mucronatum*.

The woody soil seedbank community was significantly different among sites (pseudo-F = 4.83, p < 0.001). The CAP ordination showed that height had no significant effect on the woody soil seedbank community (pseudo-F = 0.70, p = 0.784). After combining distances within transects, higher order effects were analyzed (see materials and methods). The analysis of the hierarchical design of surface flow permanence (temporary vs. permanent streams) and land use (natural vegetation vs. crop areas) with PERMANOVA analyses showed that none of the factors was statistically significant (pseudo-F = 0.35, p = 0.902; pseudo-F = 0.98, p = 0.425; respectively); neither was the interaction term land use × surface flow permanence (pseudo-F = 0.95, p = 0.418). In contrast there was a significant effect of season (pseudo-F = 11.30, p = 0.001; Fig 3); but not of the interaction term surface flow permanence x season (pseudo-F = 1.67, p = 0.107).

In order to achieve a better knowledge on woody soil seedbank community processes under natural vegetation and crop areas we performed separated statistical analyses on each land use levels. In natural vegetation areas the CAP ordination analysis showed no significant effect of height (pseudo-F = 0.62, p = 0.788). PERMANOVA analyses indicated no significant effects of surface flow permanence on woody seedbank communities (pseudo-F = 1.12, p = 0.506). In contrast there was a significant effect of season (pseudo-F = 5.39, p = 0.002) and a marginal significant effect of the interaction term surface flow permanence × season (pseudo-F = 1.93, p = 0.054). In cultivated areas PERMANOVA analyses showed no significant effect of surface flow permanence (pseudo-F = 0.33, p = 0.980); neither of season (pseudo-F = 11.68, p = 0.120) on woody seedbank communities. In contrast there was a significant effect of the interaction term surface flow permanence x season (pseudo-F = 4.25, p = 0.025) on woody seedbank communities.

The indicator species analysis showed that *Taxodium mucronatum* is a representative species of riparian woody soil seedbanks in dry seasons (IndVal: 0.85, P = 0.003). We did not find statistically significant indicator species for land use or surface flow permanence factors.

#### Effects on the herbaceous soil seed bank assemblage

**Fig 4:**
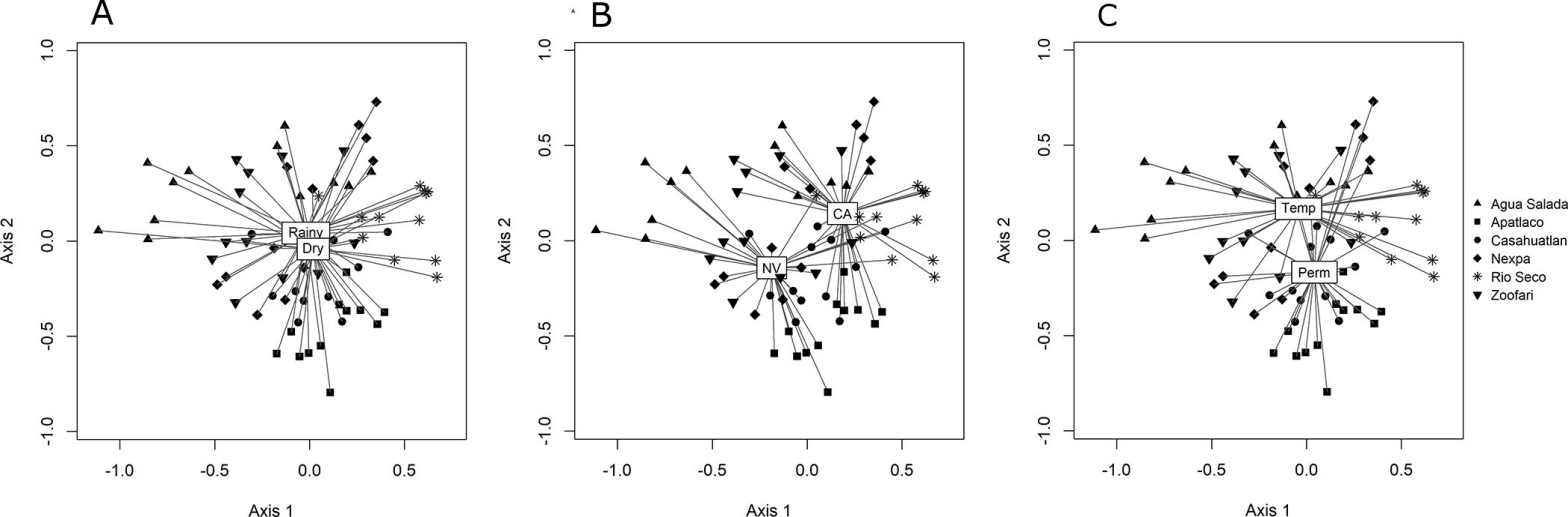
Spatial heterogeneity of the herbaceous species assembly in the soil seedbank. Significant differences between sites and the effect of season: Rainy/Dry (A); land use: natural vegetation (NV) and crop areas (CA) (B), and surface flow permanence category: temporary (Temp) and permanent (Perm) streams (C).

The herbaceous soil seedbank community was significantly different among sites (pseudo-F = 3.74, p = 0.013). The CAP analysis showed that height above river level had a significant effect on the seed community of herbaceous species in the soil seedbank (pseudo-F = 1.83, p = 0.026). After combining distances within transects, higher order effects were analyzed (see materials and methods). All terms were statistically significant, season (pseudo-F = 3.59, p < 0.001; Fig 4A), land use: natural vegetation vs. crop areas (pseudo-F = 5.29, p < 0.001; Fig 4B), surface flow permanence: temporary vs. permanent streams (pseudo-F = 6.37, p = 0.002; Fig 4C), and the interaction term land use × surface flow permanence (pseudo-F = 2.35, p = 0.002). In contrast, the interaction term surface flow permanence × season was not statistically significant (pseudo-F = 1.05, p = 0.391). We report that the permutational analysis of dispersion was significant for the factor surface flow permanence (betadisper pseudo-F = 11.52, p = 0.001), indicating unequal variances between permanent and temporal rivers. However, trends are visible in the NMDS plot, as herbaceous soil seedbank community samples were more tightly clustered in permanent compared to temporal rivers.

Separated statistical analyses on each land use level showed that in natural vegetation areas the herbaceous community in soil seedbanks was affected by height as has been shown by CAP ordination (pseudo-F = 1.79, p = 0.013), contrasting with the result of the woody community in soil seedbanks. Additionally, PERMANOVA analysis showed significant effects of surface flow permanence and season on the herbaceous seed assemblage in soil seedbanks (pseudo-F = 4.62, p = 0.006 and pseudo-F = 2.98, p < 0.001, respectively), but not surface flow permanence x season (pseudo-F = 0.76, p = 0.821). In cultivated areas there were significant effects of surface flow permanence (pseudo-F = 3.43, p < 0.044), season (pseudo-F = 3.41, p < 0.001) and the interaction term surface flow permanence x season (pseudo-F = 1.63, p = 0.025).

The indicator species analysis showed that *Polygonum tomentosum*, *Commelina diffusa* and *Desmodium sericophyllum* were characteristic of natural vegetation areas, and that *Cyperus aggregatus*, followed by *Cyperus iria* and *Parthenium hysterophorus* were the most distinctive to permanent streams (additional species are listed in Table 2).

**Table 2:** List of representative herbaceous species in the riparian soil seedbank resulting from the Indicator Species Analysis; comparison between natural vegetation and crop areas, and permanent and temporary streams.

Condition	Species	IndVal	P
Natural vegetation area	*Polygonum tomentosum* Willd.	0.788	0.01
	*Commelina diffusa* Burm. f.	0.853	0.029
	*Desmodium sericophyllum* Schltdl.	0.729	0.044
Permanent rivers	*Cyperus aggregatus* (Willd.) Endl.	0.997	0.001
	*Cyperus iria* L	0.979	0.001
	*Parthenium hysterophorus* L.	0.912	0.001
	*Conyza filaginoides* (DC.) Hieron.	0.905	0.001
	*Amaranthus spinosus* L.	0.848	0.034
	*Polygonum acuminatum* Kunth.	0.816	0.002
	*Plantago major* L.	0.774	0.011
	*Samolus ebracteatus* Kunth.	0.764	0.007
	*Verbena Carolina* L.	0.763	0.005
	*Polygonum tomentosum* Willd.	0.75	0.025
	*Chenopodium ambrosioides* L. (W. A. Weber).	0.728	0.014
	*Mercadonia procumbens* Novara, L. J. & F. C. Juárez.	0.722	0.028
	*Diplotaxis muralis* (L.) DC.	0.699	0.034
	*Eclipta prostrata* (L.) L.	0.682	0.031
	*Solanum americanum* Mill.	0.655	0.044

#### Effects of physical environmental variables on soil seed abundances

**Fig 5:**
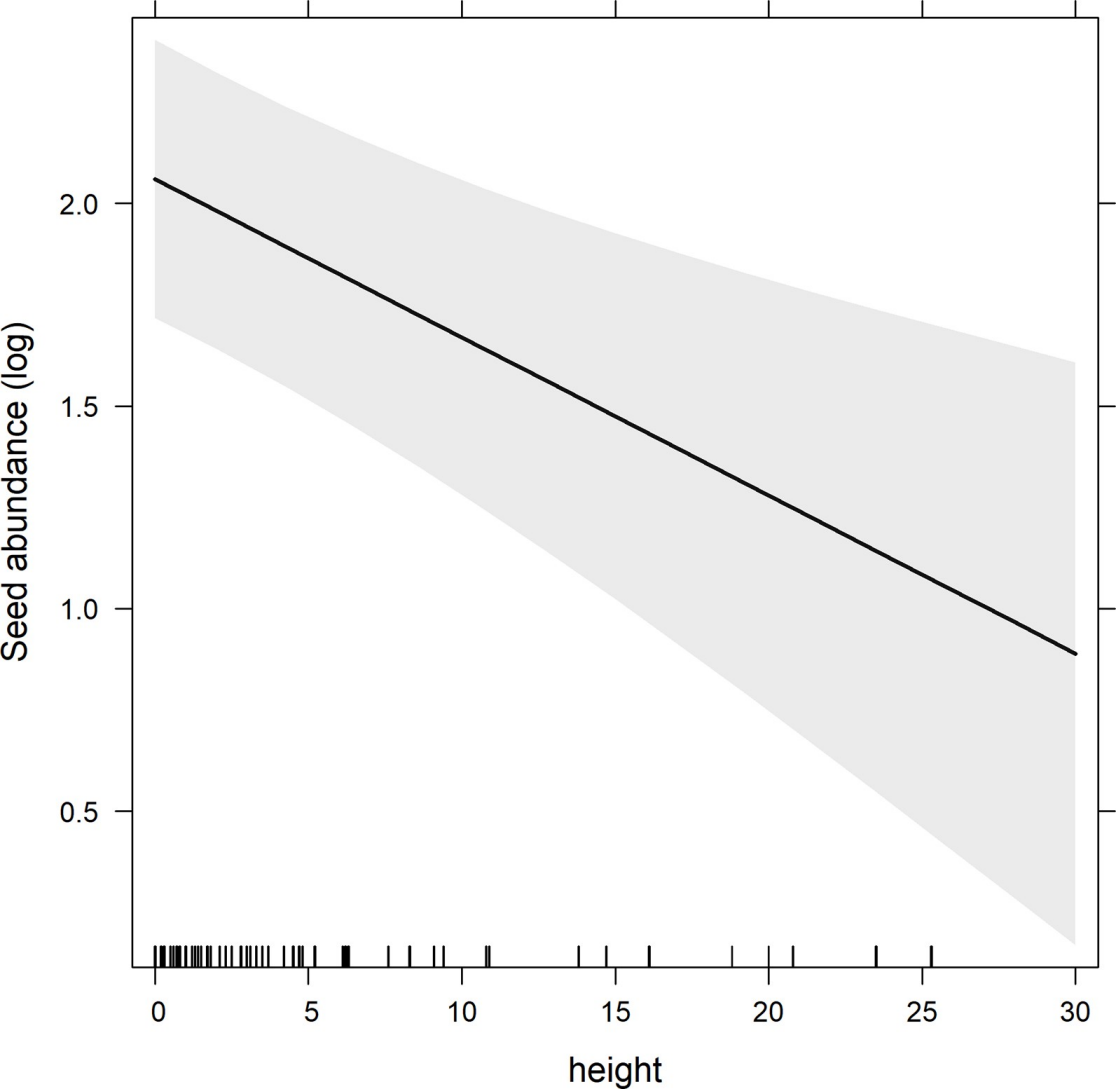
Relationship between slope and seed abundance of herbaceous and tree species in the soil seedbank of tributary streams to the Amacuzac River, Morelos. Shaded areas represent the 95% confidence limits.

Mixed-model analysis showed significant effects of year, height, season, surface flow permanence and of the land use x surface flow permanence interaction term. However, no significant results were found for the two-way interaction terms, season x height, surface flow permanence x height, season x land use, season x surface flow permanence, and the three-way interaction season x surface flow permanence x land use (Table 3). We found a negative relationship between height and seed abundance (Fig 5). Because land use history may alter ecological processes significantly we proceed to perform extended separated statistical analyses on each land use level in order to identify possible impacts of key environmental factors on plant community abundances in the natural and agricultural areas and thus, better understand seedbank processes.

**Table 3:** Results of the mixed-model analysis used to assess the effects of physical environmental variables on total seed abundances in riparian SSB in two consecutive years. Significant effects are highlighted in bold.

Effect	df	F	P
Year	1, 6	6.97	**0.0385**
Height (h)	1, 132	14.47	**< 0.001**
Season (Se)	1, 648	42.30	**< 0.001**
Surface flow permanence (SFP)	1, 6	10.23	**0.019**
Land use (LU)	1, 35	0.09	0.772
SFP x LU	1, 30	6.18	**0.019**
H x Se ^(1)^	1, 648	3.36	0.0672
H x SFP ^(1)^	1, 123	0.36	0.550
Se x LU ^(1)^	1, 648	0.79	0.375
SFP x Se ^(1)^	1, 648	0.39	0.530
Se x SFP x LU ^(1)^	1, 648	0.003	0.957

^(1)^ Terms dropped from the full model

In natural vegetation areas mixed-model analysis showed significant effects of year, height, season, surface flow permanence, and the height x season interaction term on the seed abundance in the soil seedbank. However, no significant result was found for the surface flow permanence x season and height x surface flow permanence interaction terms on seed abundance (Table 4). The significant result of the height x season interaction was due to a steeper negative relationship between height and seed abundance during the dry season than during the rainy season. In contrast, model fitted on the seed abundance from crop areas (without incorporating the covariable height, because these corresponded to flat areas) showed only two factors that were statistically significant, season and surface flow permanence. Additionally, we found no significant variation between years and of the interaction term surface flow permanence x season (Table 5) in crop areas.

**Table 4:** Results of the mixed-model analysis used to assess the effects of physical environmental variables on total seed abundances in riparian soil seedbanks from natural vegetation areas in two consecutive years. Significant effects are highlighted in bold.

Effect	df	F	P
Year	1, 18	8.58	**0.009**
Height (H)	1, 21	9.20	**0.003**
Season (Se)	1, 336	27.01	**< 0.001**
Surface flow permanence (SFP)	1, 6	8.72	**0.027**
H x Se	1, 324	4.37	**0.037**
SFP x Se ^(1)^	1, 336	0.18	0.675
H x SFP ^(1)^	1, 203	2.92	0.089

^(1)^ Terms dropped from the full model

**Table 5:** Results of the mixed-model analysis used to assess the effects of physical environmental variables on total seed abundances in riparian soil seedbanks from crop areas in two consecutive years. Significant effects are highlighted in bold.

Effect	df	F	P
Season (Se)	1, 336	19.91	**< 0.001**
Surface flow permanence (SFP)	1, 6	8.30	**0.028**
Year	1, 6	4.43	0.080
SFP x Se ^(1)^	1, 336	0.22	0.638

^(1)^ Term dropped from the full model

## Discussion

Vegetation communities in tropical dry forests riparian corridors of the Amacuzac River basin showed a clear pattern of spatial differentiation in the standing woody vegetation community as well as the tree and herbaceous components in the soil seedbank. This pattern was consistent with the great spatial heterogeneity inherent of tropical dry forests in Mexico [30]. Our results do not support the suggested homogenizing effect of rivers on riparian plant assemblages (the effects of floods as a process that reduce spatial variability) [83,84,85,86,87], but rather with the results of Esper-Reyes et al. [58], which showed varied species assemblages and seed abundance dispersed by the Amacuzac river throughout the year. We also found that other key physical/environmental variables and factors such as, land use, surface flow permanence, season, height or elevation above river, and year had significant influences on riparian species assemblages and/or seed abundances in both the herbaceous and tree components of the riparian germinable soil seed bank. Thus, according to [88], our results suggest that the high spatial differentiation of riparian vegetation assemblages may be a consequence of several key physical/environmental conditions that may be acting together but differently on the standing woody vegetation and the soil seed bank, creating diverse niches for plant species and at the same time, that the expression of this variability occurs preferably in areas with natural riparian vegetation.

### Effects of physical environmental variables on standing vegetation

Elevation above river (height) calculated from the slope and the distance to the river provides a relevant proxy of water availability and flooding stress for plants and allows comparisons with previous results that used this metric on riparian plant communities [e.g., 60,89,90, among others]. Height was the only environmental condition affecting abundance, DBH and coverage of the standing vegetation species assemblage. Species associated with steep slopes might be affected by a gradient of soil characteristics such as water availability [91,92], nutrients [93,94,95] and soil texture [96,97]. Standing woody species may also be affected by fluvial erosion of streambanks, which could be positively associated with steepness, especially when the river reaches its maximum height. Additionally, height may affect the amount of seeds deposited by the river and safe sites for germination, and thus may potentially influence the regeneration of trees through the availability of seedlings of several species.

We found no effect of surface flow permanence on the standing vegetation species assemblage, although some studies show the contrary [60]. We expected that main differences could arise due to plants' water-stress on temporary rivers during dry seasons; however our results could indicate that many woody species in riparian tropical dry forests may rely more on subsurface and deep flows, as found in other systems [98]. On the other hand, in support of our results, we would not expect differences related with surface flow permanence if the amount of seeds deposited could have some deterministic effect, both on composition and abundance of species of the standing woody vegetation. This is because riparian corridors of temporary and permanent streams receive their main seed load during rainy seasons when both temporary and permanent streams are flowing [31,58], and therefore, the seasonal lack of water flow is not relevant for the arrival of most seeds. This mechanism is critical for vegetation community dynamics, since it avoids dispersal limitation and highlights the critical role of flooding in shaping patterns of seed deposition along the riparian corridor [44]. The interaction between surface flow permanence and height has not a key effect on standing vegetation communities, at least for abundance and DBH criteria. However, it shows a significant result for coverage. We could suppose that tree coverage is a sensitive characteristic closely related to the need to have constant humidity under soil surface [99,100,101], that in places with intermittent surface flow would be limited depending on elevation of the river level (slope and distance to the river). It has been shown that soil moisture on a catchment scale exhibits a high degree of variability in space and time and is influenced by a number of factors, such as topography [102,103] soil properties [104] and land cover/vegetation [105]. One would suppose plant distribution should be influenced by soil moisture content, and that species that inhabit communities at the edge of streams have more water availability than those far away [106]. It has been shown however that some mature trees at river edges may use little or almost no surface water, but rather extract it from deep-water strata [98]. Many plants are able to use water from the subsurface soil moisture, if it is abundant, and switch to deeper sources when the surface dries [107]. At our study sites, the water landscape is characterized by high numbers of springs suggesting that superficial flows were not the only source of water [108]; therefore, we may suggest that surface flow permanence did not limit water availability for standing vegetation or influence the riparian woody species community but depending on the height at which vegetation is located it can probably influence coverage in some species [99].

### Effects of physical environmental variables on soil seedbanks

#### Land use

The herbaceous, but not the woody composition assemblages in soil seedbanks were strongly affected by land use. The woody assemblage in cultivated sites (without trees) was similar to that of natural vegetation areas. This would indicate that dispersion either by hydrochory and/or seed shadows of woody species reaches distances that exceed the spatial scale of separation between the two land use conditions studied (cultivated vs. natural areas). Another contributing factor to non-differences could be that seeds of riparian woody species may form seedbanks [109] that persist on cultivated zones, and/or that cultivated and natural forest areas are susceptible of flooding when the river reaches its maximum height. In four of our study sites, agriculture has been continuous for at least the past 32 years; in the other two sites it has been continuous for 14 and 21 years. Our results are in contrast to those of a study carried out in tropical dry forests in Ethiopia that shows declining contribution of woody species to the soil seedbank from 5.7% after seven years to 0% after 53 years of continuous cultivation following deforestation [110]. Apparently, an increasing period of continuous cultivation following deforestation could drive the disappearance of native woody species from soil seedbanks in Ethiopia, since seed contribution may depend on tree species’ seed shadows. In our study system however, tropical dry forests riparian habitats are subjected to flooding, functioning as the main alteration factor delivering seed loads and playing a key role for successful maintenance of seed species in the soil seedbank.

The herbaceous community composition, on the other hand, was different between crop and natural vegetation areas. This can be explained by the great abundance of reproductive herbaceous plants, mainly exotic, that are characteristic of disturbed-cultivated sites [110]. We attribute the high herbaceous species richness found in soil seedbanks at our study sites to the varied agroecosystems developed on the land traversed by the Amacuzac River tributaries. For example, the expansion of rice production into the region [111], is commonly related to high numbers of exotic herbaceous species. Several studies show that the invasion of exotic species associated with anthropogenic disturbance increases exotic propagule input [50,112,113]. This pattern of dominance by non-native species is common to many riparian systems in the world [50,114,115] and represents a challenge for the conservation of tropical dry forests biodiversity. As a consequence, in many countries active removal or control of exotic species has been recently proposed as an important component of river management and restoration activities [e.g. 46,116,117]; however long-term monitoring of control methods of target invasive species is necessary to implement in order to avoid secondary invasions of non-target weeds in riparian zones [118]. Riparian vegetation in Mexican tropical dry forests is not an exception to that pattern. In contrast to other more developed economies in the world, Mexico has a rich tradition of ethnobotanical knowledge that includes non-native plants as edible and medicinal resources, providing an important livelihood to local inhabitants [119, 120]. This resource will be threatened if non-native plants are eradicated or limited, a controversial issue that needs further development for the design of conservation policy in this country.

Land use history has the potential to alter ecological processes thus, separated analyses in the natural and agricultural areas of the effects of environmental variables on the woody and herbaceous seed assemblages may provide a better understanding on seedbank processes. In the general analysis, season showed a significant effect on both, the woody and the herbaceous assemblages in soil seedbanks; however in the extended analysis we found that season had no significant effect on the woody assemblage of cultivated areas. The uniformity of the woody assemblage in the soil seedbank of crop areas along the year may be resulting from river overflows, as we have mentioned before. The river can easily penetrate these areas since they do not have steep slopes. This does not seem to occur with the woody and the herbaceous species assemblage in the soil seedbank of natural vegetation areas or with the herbaceous assemblage of crop areas, for which the seasonality effect is significant. Probably, this may be resulting from a seasonal dispersion of herbs and by the particular management practices in crop areas; on the other hand, natural vegetation areas may be functioning as filters for the arrival and accumulation of seeds in general. Stream riparian zones are characterized by steep hydrological gradients likely to promote environmental filtering, and by spatiotemporal variation in the arrival of propagules likely to promote dispersal filtering. Both have been shown to be important determinant of species distributions and vegetation patterns along early successional riparian gradients [121].

The other contrasting result with the general analysis was that the interaction term surface flow permanence × season had not shown significant results neither for the woody, nor for the herbaceous component of soil seedbanks. Similarly, in terms of seed abundance in soil seedbanks, this interaction did not present significant effects either in natural vegetation or in crop areas. Contrasting, when analysis is separated according to the type of land use, there appears significant effects of the interaction term either, in woody seed assemblages of natural vegetation and crop areas, and in the herbaceous seed assemblage of crop areas. Apparently, assemblages of seed species will vary in soil seedbanks whether it corresponds to an intermittent stream in dry or rainy season, or whether it is a permanent stream in dry or rainy season. In addition to this variability, species assemblages in crop areas must also be responding to the types of management carried out in them. Respecting tree assemblages, this could be an important aspect in terms of vegetation management or restoration practices that can be done according to the season and the type of tributary river considered. However, the interaction between surface flow permanence and season had no effect on the herbaceous assemblage in soil seedbanks of natural vegetation areas, in other words, there is no dispersal or environmental filtering for the widely distributed herbs in riparian natural vegetation [121].

#### Surface flow permanence

We did not find differences in the woody composition in the soil seedbank related to surface flow permanence, which can be explained −as was already mentioned above for standing vegetation− by the fact that in these riparian corridors the main seed dispersal period occurs during rainy seasons for both temporary and permanent streams [31]. In contrast, the herbaceous communities in the soil seedbank were sensitive to the permanent vs. temporary character of streams. Herbaceous species rapidly colonize the shorelines because they are commonly water restricted; therefore, they prefer environments with permanent water presence [122]. Due to the fast growth and turnover rate, herbaceous species are very well adapted to these areas, which are subjected to continuous environmental changes. These results were consistent with the extended analysis. We found that surface flow permanence had important effects on total seed abundance either in natural vegetation or in cultivated areas. Because in riparian corridors seed movement is mainly lead by running water, permanent stream environments will show high seed abundances.

#### Season

Season had a marked effect on total seed abundance either, in natural vegetation and crop areas, and affected the woody and the herbaceous community composition in soil seedbanks. However, extended analyses that particularly investigated the effect of variables in natural vegetation and crop areas showed no effect of season on the woody seed assemblage of cultivated areas. Interspecific differences in tree and herb dispersion phenology in Mexican tropical dry forests [123] and in the study region [124] may influence seed composition and numbers that would be available in the environment. Commonly, in this mexican tropical dry forests matrix tree species show a marked seed dispersal peak during dry seasons [123,125] which is related to windy seasons; however the river showed differential seed dispersal composition and abundance in this region [58] that was greater during the rainy seasons than in the dry ones. As mentioned above (Land use section) when rivers overflow spread seed communities that will accumulate in the soil seedbank. Seeds of herbaceous, pioneer species and principally exotic species, in contrast to woody species generally have small seeds that growth fast in open conditions [126,127]; they also are adapted to tolerate drought [128], and therefore, season may have a more evident effect on herbaceous than on woody seed assemblages.

The tree *Taxodium mucronatum* was the most representative species along tropical dry forests riparian corridors in Morelos, and we found that it was the most representative in the soil seedbank during dry seasons. Esper-Reyes et al’s [58] results indicated that seeds of *T*. *mucronatum* were always present in the environment and dispersed by the river during rainy and dry seasons. In contrast, it has been shown that in the U.S.A., the closely related species *T*. *distichum* (L.) L.C. Rich, is sensitive to climatic and hydrological changes [129,130,131,132,133], and to the effects of stream channelization, inhibiting population maintenance [134].

#### Height

We found a marked effect of height on total seed abundance and on the herbaceous assemblage composition, but not on that of the woody assemblage in the soil seedbank. The herbaceous assemblage composition was sensitive to height; herbaceous species rapidly colonize humid shorelines that allow them to grow quickly in areas which are subjected to continuous changes and impacts. The lack of height effect on the woody assemblage composition may be a consequence of the river’s potential to completely inundate the riparian corridor. Riis et al [135] mentioned that depending on the elevation and the distance to the river, there would be two mechanisms that work in opposite ways; near the river we would expect that greater water flow would result in greater quantity of seeds deposited on the shorelines, but this effect could be counteracted by higher current velocity causing a shear stress and the removal of most seeds from these sites. The presence of these two mechanisms may result in no changes of the composition and abundance of species along the distance/height gradient.

We also found an important season × height interaction effect on seed abundance in natural vegetation areas, since crop areas do not present topographical slopes. Total seed abundances in the soil seedbank were higher during dry seasons, in permanent streams and at low height values, when compared with other environmental conditions. The effect of steepness depended on season; height was very important in dry seasons, but not in rainy seasons; and in natural vegetation areas. In this ever-changing riparian environment, dry seasons represent periods of settlement, when soil seedbanks are full of seeds brought and dispersed by rivers during rainy seasons, and it is at this period when the effect of the elevation above the river level is evident in total seed numbers. This result also suggests that in rainy seasons, river flow levels can reach the higher parts of riparian corridors, dragging seed loads with it. In general, most seeds were deposited along the shores, where lower steepness allows inundation, and this is evident for dry seasons. Contrastingly, the effect of height above river level is independent of whether the river is temporary or permanent (superficial flow permanence) on seed abundances in the soil seedbank.

We also found that total seed abundances varied between the two years of the study. We suggest this may be the result of any process related to inter-annual plant phenology variation, as well as to inter-annual river flow variations, both of which may be affected by a complex topography and the heterogeneous distribution of rains at regional scales. The temporal and spatial variability of species abundance and composition of the riparian vegetation soil seedbank could be a main factor that explains the extraordinarily high alfa and beta diversity of Mesoamerican tropical dry forests [136], in which riparian vegetation is immersed. In crop areas the effect of year differences on seed abundance in soil seedbanks is not evident and this may be a result of management practices on these areas.

Geomorphologically suitable for crop establishment and agricultural developments, tropical dry forests riparian vegetation covers the discharge area of many rivers constituting the most productive landscape in the studied region [137], and have a long history of changes in land use mainly related to the sugarcane production [138]. Therefore, common problems have historically involved high anthropogenic pressure and natural disasters of overflowing rivers [21,139]. This is also evidenced from the representative herbaceous species in the riparian soil seedbanks resulting from the indicator species analysis in this study, in which three non-native species- *Polygonum tomentosum*, *Commelina diffusa* and *Desmodium sericophyllum-* were representative of natural vegetation areas. The seeds of *P*. *tomentosum* are characteristic for having a short viability time [140], so they are viable in open spaces and need enough light to germinate [141]. Likewise, *C*. *diffusa* is an indicator plant of cultivated and humid areas and *D*. *sericophyllum* is an herbaceous plant associated with rice crops in Vietnam [142]. These herbaceous species seem to thrive well in half-shade humid environments. Some of these species have been found associated with forests and even sub-deciduous forests in ravines and therefore we assume that they receive less light [143]. Indicator species of permanent rivers were common seed species found on the banks of irrigation channels and drainage, ruderal sites, in croplands with irrigation and in general, associated with marshes and wetlands [144,145,146,147].

Finally, we want to acknowledge some potential limits of this study, for example, the seedling emergence method used of 3-month period captures the germinable seed bank, that is, those species that readily germinate in response to favourable conditions. Thus, this method may fail to detect those species with long-term physical or chemical dormancy. We think our results show an interesting perspective about the environmental drivers of riparian vegetation and seedbanks in tropical systems, commonly less studied than boreal and temperate ones. This may contribute to define innovative strategies for their conservation.

## Conclusions

In contrast to standing vegetation, soil seedbank communities along tributary streams of tropical dry forests were vulnerable to distinct environmental factors such as seasonal variations, surface flow permanence, height and anthropogenic alteration [60]. Our results, highlight that both the composition and the abundance of the herbaceous assemblage in soil seedbanks have an important indicator value for determining the conservation status of riparian vegetation along tributaries of tropical dry forests. Our results also suggest that in terms of seed abundances most variables evaluated are evident in natural vegetation areas that provide spatial variability, therefore we highlight the conservation value that stress the need to maintain these areas.

Trees are considered the most important elements in riparian ecosystems [141], providing landscape diversity [148,149] and playing an important role in the dynamics of wetland ecotones [150]. It has been argued that understanding the dynamics of long-lived trees that function as “engineer species” in riparian or xero-riparian environments provides a solid basis for ecosystem management [151]. In our study, only 40% of the woody component in soil seedbanks was represented in the standing vegetation, which is a common pattern reported in several studies [36, 110,152,153]. The herbaceous community dominated soil seedbanks at all sites in terms of abundance and richness; in particular, soil seedbanks of cultivated areas did not differed in abundance from natural vegetation areas, but they differed in community composition, and in the effect that some variables and factors have on their soil seedbanks.

Increasing attention and knowledge on vegetation dynamics at tributary rivers is an important element required for successful restoration efforts and for the placement of riparian buffers that could reduce nutrient losses to streams [153]. Herbaceous communities are of ecological relevance for riparian restoration initiatives; they are abundant, have short life cycles and are mostly considered pioneer species [154]. Their primary function is the protection of bare soils, preventing soil erosion by water flows and wind [155]. It has been argued that directed succession, a common restoration tool in a number of human-modified habitats, may be less efficient in riparian zones because they are dynamic in nature and suffer from continuous propagule pressure from non-native species [50]. Restoration of riparian corridors should not attempt to reconstruct an ecosystem by returning it to some previous condition, which is mostly unknown in riparian habitats, but rather to maintain river health and key ecosystem services [50]. Thus, herbaceous plants may play a significant role in the promotion and establishment of new seedlings by facilitating this procedure and by fixing sediments on river banks, therefore increasing soil organic matter [151].

The consistent spatial heterogeneity pattern found in vegetation dynamics characteristic of the Amacuzac River tributaries suggests that the environmental local conditions identified in this study are critical factors that must be considered for any management and conservation strategy to be implemented in river corridors and highlights the conservation values of temporary and permanent tributary rivers in the region. The finding of this ubiquitous character allows us to suppose the constant creation and availability of diverse ecological niches along riparian tibutaries for plant species, and this may contribute to define innovative strategies for their conservation and restoration. This study provides evidence that both, at a regional and local scale, such as topographic variations, abiotic factors correlate with variations in plant biodiversity. These spatial variations are indicative of the great biodiversity of tropical dry forests in Mesoamerica, as widely discussed in this article.

## Supporting information

S1 Table List of woody species constituting the riparian standing vegetation recorded in six tributaries to the Amacuzac River in Morelos, Mexico. Plant growth forms, tree (T) and shrub (Sh) species; species importance values (IVI), and status, native (N) or exotic (E) species.
(PDF)

S2 Table Descriptive ecological parameters of standing vegetation and soil seed bank communities in six tributaries to the Amacuzac River. (a) SV (trees and shrubs) and (b) SSB communities (trees, shrubs and herbs), in six tributaries to the Amacuzac River, considering different climatic seasons (rainy vs. dry), surface flow permanence (temporary vs. permanent), and land use (natural vegetation vs. crop areas).
(PDF)

S3 Table List of riparian vegetation communities in the soil seedbank of six tributaries to the Amacuzac River in Morelos. Plant growth form, tree (T), shrub (Sh) and herbaceous (H) species; species importance values (IVIB), and status, native (N), exotic (E), or uncertain (U).
(PDF)

S1 Fig **Species accumulation (a) and seedling abundance curves (b) over time of soil seedbanks collected along six tributaries to the Amacuzac River**, during the rainy and dry seasons of two consecutive years. Number of species and abundances were determined using the seedling emergence method under greenhouse conditions.
(PDF)

S1 File Information corresponding to the map presented in Fig 1. (PDF)

S1 Data Information on free availability of data in Fighsare repository. (PDF)
