# Supplementary material for: Physical environmental conditions determine ubiquitous spatial differentiation of standing plants and seedbanks in Neotropical riparian dry forests
Source: PLoS One. 2019 Mar 13;14(3):e0212185. doi: 10.1371/journal.pone.0212185 (PMC6415903; doi:10.1371/journal.pone.0212185)
Supplement: S1 Table — Plant growth forms, tree (T) and shrub (Sh) species; species importance values (IVI), and status, native (N) or exotic (E) species. (PDF) [file pone.0212185.s001.pdf]

Supporting information

S1 Table.

Species	Family	IVI	Growth form	Status
Ficus cotinifolia Kunth	**Moraceae**	46.25	T	N
Acacia cochliacantha Humb. & Bonpl. ex Willd	**Fabaceae**	37.92	Sh	N
Tabernaemontana litoralis Kunth	**Apocynaceae**	33.20	Sh	E
Annona squamosa L.	**Annonaceae**	24.17	T	N
Acacia riparia Kunth	**Fabaceae**	23.22	Sh	N
Acacia farnesiana (L.) Willd.	**Fabaceae**	19.64	Sh	N
Sapindus saponaria (Michú) L.	**Sapindaceae**	17.08	T	E
Prosopis laevigata (H. & B.) Jonhst	**Fabaceae**	15.89	T	N
Pithecellobium dulce (Roxb.) Benth.	**Mimoceae**	14.67	T	N
Astianthus viminalis (Kunth) Bail-lon	**Bignoniaceae**	14.21	T	N
Salix humboldtiana Willd.	**Salicaceae**	13.16	T	N
Lysiloma divaricatum (Jacq.) J.F. Macbr.	**Fabaceae**	12.88	T	N
Ricinus communis L.	**Euphorbiaceae**	12.21	Sh	E
Gliricidia sepium (Jacq.) Kunth ex Walp.	**Fabaceae**	11.45	T	N
Lysiloma acapulcensis (Kunth) Benth.	**Fabaceae**	11.37	T	N
Leucaena esculenta (Moc. & Sessé ex DC.) Benth.	**Mimoceae**	11.13	T	N
Guazuma ulmifolia Lam.	**Sterculiaceae**	10.13	T	N
Ziziphus amole (Sessé & Moc.) M.C. Johnst.	**Rhamnaceae**	10.07	T	N
Licania arborea Seem.	**Chrysobalanceae**	9.27	T	N
Daphnopsis americana (Mill.) J.R. Johnst.	**Thymelaeaceae**	9.11	T	N
Senna occidentalis (L.) Link.	**Fabaceae**	8.82	Sh	E
Inga spuria Humb. & Bonpl. ex Willd.	**Fabaceae**	8.81	T	N
Cyrtocarpa procera Kunth	**Anacardiaceae**	8.72	T	N
Cestrum tomentosum L. f.	**Solanaceae**	8.69	Sh	N
Tabebuia impetiginosa (Mart. ex DC.) Standley.	**Bignoniaceae**	8.54	T	N
Diphysa robinioides (Mill.) M.Sousa	**Fabaceae**	6.65	T	N
Piper leucophyllum (Miq.) C.DC.	**Piperaceae**	6.02	Sh	N
Swietenia humilis Zucc (caobilla).	**Meliaceae**	5.95	T	N
Leucaena leucocephala (Lam.)	**Mimoceae**	5.72	T	N
Trichilia hirta L.	**Meliaceae**	5.30	T	N
Ficus tecolutensis (Liebm.) Miq.	**Moraceae**	4.60	T	N
Lysiloma tergemina Benth.	**Fabaceae**	4.33	T	N
Bursera grandifolia Engl.	**Burceraceae**	4.33	T	N
Spondias mombin L.	**Anacardiaceae**	4.09	T	N
Senna wislizeni pringlei var. (Rose) Irwin & Barneby.	**Fabaceae**	3.78	T	N
Hamelia patens Jacq. Coralillo.	**Rubiaceae**	3.68	Sh	N
Mimosa polyantha Benth	**Fabaceae**	3.63	T	N
Bumelia obtusifolia Roem. & Schult	**Sapoteceae**	3.10	T	N

Species	Family	IVI	Growth form	Status
Alvaradoa amorphoides Liebm.	**Picramniaceae**	3.06	Sh	N
Gyrocarpus jatrophifolius Domin.	**Hernandiaceae**	2.99	T	E
Cascabela ovata (Cav.) Lippold	**Apocynaceae**	2.98	Sh	N
Ceiba pentandra (L.) Gaertn.	**Bombacaceae**	2.98	T	N
Enterolobium cyclocarpum (Jacq.) Griseb.	**Mimoceae**	2.98	T	N
Sapium macrocarpum Müll. Arg.	**Euphorbiaceae**	2.98	T	N
Haematoxylum brasiletto Karsten.	**Fabaceae**	2.98	T	N
Bunchosia canescens (Aiton) DC.	**Malpigiaceae**	2.97	Sh	N
Pisonia aculeata L.	**Nyctaginaceae**	2.97	Sh	N
Tournefortia hirsutissima L.	**Boraginaceae**	2.97	Sh	N
Heliocarpus pallidus Rose.	**Malvaceae**	2.96	T	N
Ipomea murucoides Roem. & Schult.	**Convulvulaceae**	2.96	T	N
Cedrela oaxacensis C.DC. & Rose.	**Meliaceae**	2.96	T	N
Pterocarpus orbiculatus DC.	**Fabaceae**	0.01	T	N
