# Supplementary material for: Physical environmental conditions determine ubiquitous spatial differentiation of standing plants and seedbanks in Neotropical riparian dry forests
Source: PLoS One. 2019 Mar 13;14(3):e0212185. doi: 10.1371/journal.pone.0212185 (PMC6415903; doi:10.1371/journal.pone.0212185)
Supplement: S2 Table — (a) SV (trees and shrubs) and (b) SSB communities (trees, shrubs and herbs), in six tributaries to the Amacuzac River, considering different climatic seasons (rainy vs. dry), surface flow permanence (temporary vs. permanent), and land use (natural vegetation vs. crop areas). (PDF) [file pone.0212185.s002.pdf]

Supporting information

S2 Table.

(a)

**Indices - SV**	**Temporary**	**Permanent**
Number of species	36	32
Number of individuals	146	124
Dominance (D)	0.08	0.06
Simpson (1-D)	0.92	0.94
Shannon (H')	2.97	3.10
Margalef (I)	7.02	6.43
Equitability (J)	0.83	0.90
Sorensen (Is)	0.33	

(b)

**Indices - SSB**	**Rainy**	**Dry**	**Temporary**	**Permanent**	**Natural vegetation**	**Crop areas**
Number of species	120	158	131	161	158	133
Number of individuals	3779	6254	3109	6924	5097	4936
Dominance (D)	0.06	0.03	0.03	0.06	0.05	0.04
Simpson (1-D)	0.94	0.97	0.97	0.94	0.95	0.96
Shannon (H')	3.41	3.96	3.78	3.64	3.69	3.66
Margalef (I)	14.45	17.96	16.17	18.09	18.39	15.52
Equitability (J)	0.71	0.78	0.78	0.72	0.73	0.75
Sorensen (Is)	0.81		0.89		0.77	
