# Supplementary material for: Physical environmental conditions determine ubiquitous spatial differentiation of standing plants and seedbanks in Neotropical riparian dry forests
Source: PLoS One. 2019 Mar 13;14(3):e0212185. doi: 10.1371/journal.pone.0212185 (PMC6415903; doi:10.1371/journal.pone.0212185)
Supplement: S3 Table — Plant growth form, tree (T), shrub (Sh) and herbaceous (H) species; species importance values (IVIB), and status, native (N), exotic (E), or uncertain (U). (PDF) [file pone.0212185.s003.pdf]

Supporting information

S3 Table.

Species	Family	IVIB	Growth form	Status
Cyperus aggregatus (Willd.)	**Cyperaceae**	17.41	H	N
Parthenium hysterophorus L.	**Asteraceae**	10.78	H	N
Ageratum conyzoides L.	**Asteraceae**	10.71	H	E
Bacopa sp. 2	**Plantaginaceae**	9.04	H	U
Commelina diffusa Burm. f.	**Commelinaceae**	8.05	H	N
Polypogon monspeliensis (L.) Desf.	**Poaceae**	7.64	H	E
Cyperus iria L.	**Cyperaceae**	7.52	H	E
Buddleja americana L.	**Scrophulariaceae**	7.15	H	N
Oxalis corniculata L. Agritos.	**Oxalidaceae**	6.96	H	N
Digitaria bicornis (Lam.) Roem. & Schultes.	**Poaceae**	6.01	H	E
Crusea longiflora (Willd. ex Roem. & Schult.)	**Rubiaceae**	5.65	H	N
Physalis gracilis Miers.	**Solanaceae**	4.02	H	N
Bacopa sp. 1	**Plantaginaceae**	3.92	H	U
Amaranthus hybridus L. Quintonil.	**Amaranthaceae**	3.64	H	N
Elytraria imbricata (Vahl)	**Acanthaceae**	3.08	H	N
Sagina procumbens L.	**Caryophyllaceae**	2.59	H	E
Lasiacis procerrima (Hack.) Hitchc.	**Poaceae**	2.55	H	N
Heliotropium procumbens Mill.	**Boraginaceae**	2.44	H	N
Eragrostis cilianensis (All.) Vignolo ex Janchen	**Poaceae**	2.36	H	E
Lasiacis sp. 1	**Poaceae**	2.10	H	U
Heliotropium curassavicum L.	**Boraginaceae**	1.95	H	N
Digitaria leucites (Trin.)	**Poaceae**	1.89	H	N
Ficus sp. 1	**Moraceae**	1.65	T	U
Sida rhombifolia L.	**Malvaceae**	1.55	H	U
Desmodium sericophyllum Schltdl.	**Fabaceae**	1.46	H	N
Gnaphalium cheiranthifolium Lam.	**Asteraceae**	1.32	H	N
Eleusine indica (L.) Gaertn.	**Poaceae**	1.26	H	N
Dinebra panicea (Retz.) P.M. Peterson & N.	**Poaceae**	1.26	H	N
Rorippa nasturtium-aquaticum (L.) Schinz & Thell.	**Brassicaceae**	1.18	H	E
Euphorbia hirta L.	**Euphorbiacea**	1.00	H	N
Pilea microphylla (L.) Liebm.	**Urticaceae**	0.98	H	N

Species	Family	IVIB	Growth form	Status
Conyza filaginoides (DC.) Hieron.	**Asteraceae**	0.93	H	N
Euphorbia serpens Kunth.	**Euphorbiacea**	0.93	H	E
Amaranthus spinosus L.	**Amaranthaceae**	0.90	H	E
Lobelia cordifolia Hook. & Arn.	**Campanulaceae**	0.90	H	N
Argemone mexicana L.	**Papaveraceae**	0.90	H	N
Polygonum sp. 1	**Poligonaceae**	0.87	H	U
Acacia farnesiana (L.) Willd.	**Mimosaceae**	0.82	Sh	N
Ficus sp. 3	**Moraceae**	0.81	T	U
Guazuma ulmifolia Lam.	**Malvaceae**	0.75	T	N
Diplotaxis muralis (L.) DC.	**Brassicaceae**	0.70	H	E
Portulaca oleracea L.	**Portulacaceae**	0.69	H	E
Senna uniflora (Mill.) H.S. Irwin & Barneby	**Fabaceae**	0.55	T	E
Sanvitalia procumbens Lam.	**Asteraceae**	0.50	H	N
Cyperus odoratus L.	**Cyperaceae**	0.48	H	N
Acmella repens (Walter) Rich.	**Asteraceae**	0.47	H	N
Eclipta prostrata (L.) L.	**Asteraceae**	0.47	H	N
Bacopa procumbens (Mill.) Greenm.	**Scrophulariaceae**	0.43	H	E
Drymaria villosa Cham. & Schltdl.	**Caryophyllaceae**	0.41	H	N
Apium leptophyllum (Pers.) F. Muell. ex Benth.	**Apiaceae**	0.41	H	N
Acmella oleracea (L.) R.K.Jansen	**Asteraceae**	0.41	H	E
Bidens pilosa L.	**Asteraceae**	0.39	H	N
Melampodium divaricatum (L. C. Rich.) DC.	**Asteraceae**	0.35	H	N
Crotalaria longirostrata Hook. & Arn.	**Fabaceae**	0.35	H	N
Plantago major L.	**Plantiginaceae**	0.35	H	E
Bidens odorata Cav.	**Asteraceae**	0.35	H	N
Conyza bonariensis (L.) Cronq.	**Asteraceae**	0.34	H	N
Spermacoce pusilla Wall. H	**Rubiaceae**	0.33	H	U
Diplotaxis virgata (Cav.) DC.	**Brassicaceae**	0.32	H	E
Wigandia urens (Ruiz & Pavón) Kunth.	**Hydrophyllaceae**	0.32	Sh	N
*Eragrostis pectinata (*Michx.)	**Poaceae**	0.32	H	N
Nissolia fruticosa Jacq.	**Fabaceae**	0.31	H	N
Sida Malvaceae L.	**Malvaceae**	0.29	H	U
Desmodium procumbens (Mill.) Hitchc.	**Fabaceae**	0.29	H	N

Species	Family	IVIB	Growth form	Status
Echinochloa colonum (L.) Link.	**Poaceae**	0.28	H	E
Samolus ebracteatus Kunth	**Primulaceae**	0.25	H	N
Conyza sp.1	**Asteraceae**	0.25	H	U
Solanum americana Mill., Gard. Dict.	**Solanaceae**	0.25	H	N
Bacopa sp. 3	**Plantaginaceae**	0.23	H	U
Stemodia durantifolia (L.) Sw.	**Scrophulariaceae**	0.21	H	N
Eleocharis geniculata (L.) Roem. & Schult.	**Cyperaceae**	0.20	H	E
Ficus sp. 2	**Moraceae**	0.20	T	U
Acmella radicans (Jacq.) R. K. Jansen.	**Asteraceae**	0.19	H	N
Ipomoea murucoides Roem. & Schult.	**Convolvulaceae**	0.19	T	N
Mimulus glabratus Kunth.	**Phrymaceae**	0.19	H	N
Chenopodium ambrosioides L. (W. A. Weber).	**Chenopodiaceae**	0.18	H	N
Dalea sp. 1	**Fabaceae**	0.18	H	U
Mentzelia hispida Willd.	**Loasaceae**	0.18	H	N
Paspalum notatum Flüggé	**Poaceae**	0.17	H	N
Oenothera rosea L´Hér. ex Ait.	**Onagraceae**	0.17	H	N
Spermacoce ocymoides L.	**Rubiaceae**	0.17	H	E
Crotalaria pumila Ort.	**Fabaceae**	0.16	H	N
Acalypha arvensis Poepp. & Endl.	**Euphorbiacea**	0.16	H	N
Delilia biflora Spreng.	**Asteraceae**	0.16	H	N
Bellis perennis L.	**Asteraceae**	0.14	H	E
Argemone ochroleuca Sweet.	**Papaveraceae**	0.14	H	N
Solanum sp. 1	**Solanaceae**	0.14	H	U
Dalea foliolosa (Ait.) Barneby.	**Fabaceae**	0.14	H	N
Ipomoea purpurea (L.) Roth.	**Convolvulaceae**	0.13	H	N
Nicotiana glauca Graham	**Solanaceae**	0.12	Sh	E
Celtis caudata Planchon	**Ulmaceae**	0.11	T	N
Oxalis alpina (Rose) R. Knuth.	**Oxalidaceae**	0.11	H	N
Portulaca pilosa L.	**Portulacaceae**	0.11	H	N
Taraxacum obovatum (Willd.) DC.	**Asteraceae**	0.11	H	N
Lobelia cardinalis L.	**Campanulaceae**	0.11	H	N
Tithonia tubaeformis (Jacq.) Cass.	**Asteraceae**	0.11	H	N
Opuntia decumbens Salm-Dyck.	**Cactaceae**	0.10	H	N

Species	Family	IVIB	Growth form	Status
Tagetes lucida Cav.	**Asteraceae**	0.10	H	N
Asterohyptis stellulata (Benth.) Epling	**Lamiaceae**	0.10	Sh	N
Euphorbia sp. 1	**Euphorbiacea**	0.09	H	U
Verbena carolina L.	**Verbenaceae**	0.09	H	N
Oplismenus undulatifolius (Ard.) P. Beauv.	**Poaceae**	0.09	H	E
Taxodium mucronatum Ten.	**Cupressaceae**	0.08	T	N
Conyza sophiifolia Kunth.	**Asteraceae**	0.08	H	N
Polygonum tomentosum Willd.	**Poligonaceae**	0.08	H	N
Indigofera cuernavacana Rose	**Fabaceae**	0.08	Sh	N
Cleome chilensis DC.	**Capparaceae**	0.08	H	N
Asplenium trichomanes L.	**Aspleniaceae**	0.07	H	E
Solanum sp. 3	**Solanaceae**	0.07	H	U
Bidens bigelovii A. Gray.	**Asteraceae**	0.06	H	N
Conyza canadensis (L.) Cronquist.	**Asteraceae**	0.06	H	N
Euphorbia sp. 2	**Euphorbiacea**	0.06	H	U
Solanum sp. 2	**Solanaceae**	0.06	H	U
Aeschynomene americana L.	**Fabaceae**	0.06	H	N
Cenchrus pauciflorus Benth.	**Poaceae**	0.06	H	N
Euphorbia sp. 3	**Euphorbiaceae**	0.06	H	U
Tibouchina pringlei Rose.	**Melastomaceae**	0.06	H	E
Polygonum acuminatum Kunth.	**Poligonaceae**	0.05	H	N
Hyptis suaveolens (L.) Poit.	**Lamiaceae**	0.05	H	N
Acmella sp.1	**Asteraceae**	0.05	H	E
Bacopa monnieri (L.) Wettst.	**Plantaginaceae**	0.05	H	E
Lobelias sp.1	**Campanulaceae**	0.04	H	U
Urtica dioica L.	**Urticaceae**	0.04	H	N
Bursera copallifera (D.C.) Bullock	**Burseraceae**	0.03	T	N
Ficus sp. 4	**Moraceae**	0.03	T	U
Panicum trichoides Sw.	**Poaceae**	0.03	H	N
Pteris vittata L.	**Pteridaceae**	0.03	H	E
Ipomoea pauciflora M. Martens & Galeotti	**Convolvulaceae**	0.03	T	N
Cyperus rotundus L.	**Cyperaceae**	0.03	H	E
Desmodium tortuosum (Sw.) DC.	**Fabaceae**	0.03	H	E

Species	Family	IVIB	Growth form	Status
Sonchus oleraceus L.	**Asteraceae**	0.03	H	E
Spermacoce assurgens Ruiz & Pav.	**Rubiaceae**	0.02	H	N
Datura stramonium L.	**Solanaceae**	0.02	H	N
Cuphea aequipetala Cav.	**Lythraceae**	0.02	H	N
Evolvulus alsinoides (L.) L.	**Convolvulaceae**	0.02	H	N
Kallstroemia rosei Rydb.	**Zygophyllaceae**	0.02	H	N
Oenothera biennis L.	**Onagraceae**	0.02	H	N
Sonchus arvensis L.	**Asteraceae**	0.02	H	N
