# Supplementary figures and images for: Physical environmental conditions determine ubiquitous spatial differentiation of standing plants and seedbanks in Neotropical riparian dry forests
Source: PLoS One. 2019 Mar 13;14(3):e0212185. doi: 10.1371/journal.pone.0212185 (PMC6415903; doi:10.1371/journal.pone.0212185)

## Supporting information

S1 Fig.

(a)

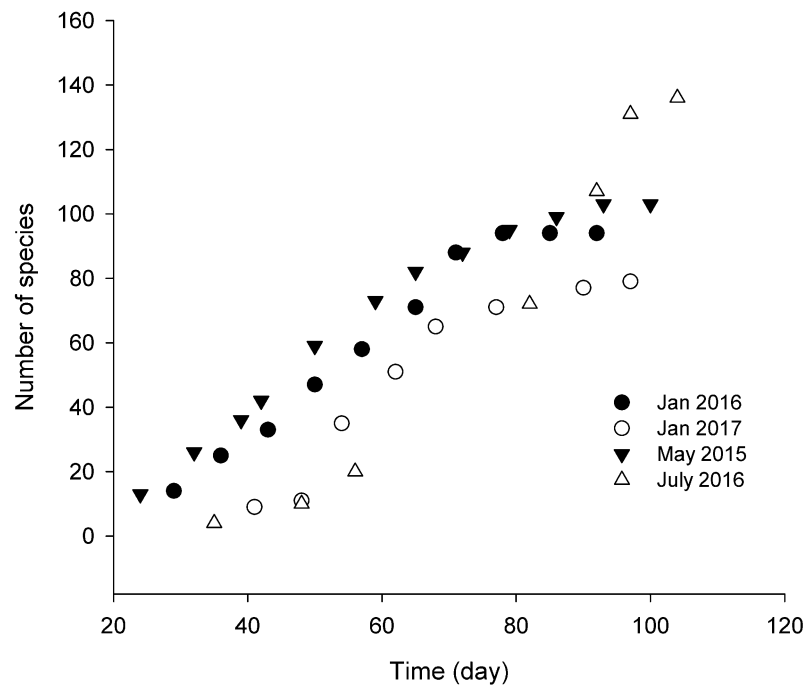

(b)

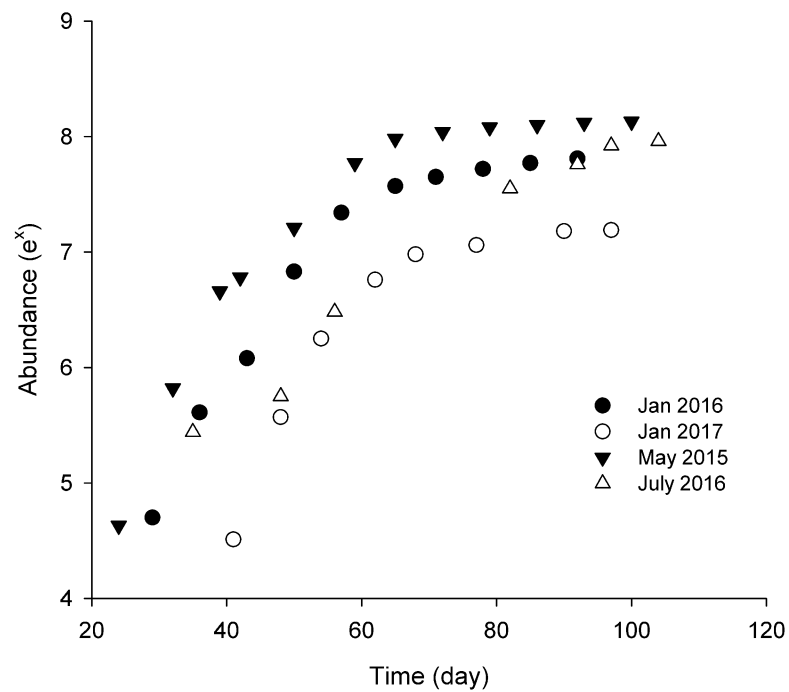

Supplement: S1 Fig — Species accumulation (a) and seedling abundance curves (b) over time of soil seedbanks collected along six tributaries to the Amacuzac River, during the rainy and dry seasons of two consecutive years. Number of species and abundances were determined using the seedling emergence method under greenhouse conditions. (PDF) [file pone.0212185.s004.pdf]
