# Supplementary material for: Physical environmental conditions determine ubiquitous spatial differentiation of standing plants and seedbanks in Neotropical riparian dry forests
Source: PLoS One. 2019 Mar 13;14(3):e0212185. doi: 10.1371/journal.pone.0212185 (PMC6415903; doi:10.1371/journal.pone.0212185)
Supplement: S1 File — (PDF) [file pone.0212185.s005.pdf]

Supporting information

S1 File. The map presented in Figure 1 was modified by authors using the information layers accessed from the following link: https://www.inegi.org.mx/app/geo2/ntm/, at INEGI National Institute of Statistics and Geography (Mexico) website.

Data Sets within the framework of the Public Service of Statistical and Geographical Information, are made available as Open Data, with the purpose of facilitating their access, use, consultation, reuse and redistribution for any purpose, all for free according to what is established in the "Terms of Free Use of INEGI Information", published on the INEGI Website, which can be consulted in the link: http://www.beta.inegi.org.mx/inegi/terminos.html.

NOTICE TO USER

To use the information from Instituto Nacional de Estadística y Geografía / National Institute of Statistics and Geography (INEGI), it constitutes acceptance of the following

FREE USE OF INFORMATION FROM INEGI

1. **The user:**
   a. Can make and distribute copies of the information, without altering or deleting metadata.
   b. Can disseminate and publish the information.
   c. Can adapt and reorder the information.
   d. Can extract all or part of the information.
   e. Can exploit the information commercially, using it as an input to produce other products or services
   f. INEGI must be given as author, and if technically possible, mention the source of information extraction as follows: "Source: **INEGI**, product name from which information is extracted" and if applicable update date, e.g. "Source: **INEGI**, 2009 Economic Censuses".
   g. Should not use the information in order to mislead or confuse the population varying the original sense of it and its veracity.
   h. Should not pretend that the use of information, represents an official position of **INEGI** or that it is endorsed, integrated, sponsored or supported by the source.
2. **INEGI** is not responsible for the users' interpretation and application of the results obtained through the use of information; so any decision based on their interpretation excludes **INEGI** of any responsibility. Likewise, **INEGI** will not be responsible for the differences obtained by precision, rounding or truncation errors, as well as technical or technological changes that could affect such results.
3. The validity of these Terms of Free Use is for indefinite time as long as the above provisions are not convened.
4. Unauthorized use contrary to these Terms of Free Use, shall be punished in accordance with applicable law.
5. These Terms of Free Use are governed by the Law of the National System of Statistical and Geographic Information, as well as by the regulations applicable in the matter, if any controversy, the user agrees to submit to the jurisdiction and competency of the Federal Courts of Mexico City, Distrito Federal, waiving the jurisdiction that may correspond by reason of current or future domicile or otherwise.
6. **INEGI** reserves the right to modify at any time these Terms of Free Use.
