# Supplementary material for: Physical environmental conditions determine ubiquitous spatial differentiation of standing plants and seedbanks in Neotropical riparian dry forests
Source: PLoS One. 2019 Mar 13;14(3):e0212185. doi: 10.1371/journal.pone.0212185 (PMC6415903; doi:10.1371/journal.pone.0212185)
Supplement: S1 Data — (PDF) [file pone.0212185.s006.pdf]

Supporting information

S1 Data.

All data underlying the study are available on the Figshare repository at https://figshare.com/s/b878268371261dc5dff2. DOI: 10.6084/m9.figshare.7390523
